# Plasma for Energy and Catalytic Nanomaterials

**DOI:** 10.3390/nano10020333

**Published:** 2020-02-15

**Authors:** Feng Yu, Lanbo Di

**Affiliations:** 1 Key Laboratory for Green Processing of Chemical Engineering of Xinjiang Bingtuan, School of Chemistry and Chemical Engineering, Shihezi University, Shihezi 832003, China; 2 Bingtuan Industrial Technology Research Institute, Shihezi University, Shihezi 832003, China; 3 College of Physical Science and Technology, Dalian University, Dalian 116622, China

This Special Issue “Plasma for Energy and Catalytic Nanomaterials” of Nanomaterials is focused on advancements in synthesis and applications of energy and catalytic nanomaterials by plasma. The preparation of nanomaterials is gaining increasing interest for energy and catalytic applications, such as methane reforming, Fischer-Tropsch synthesis, an oxygen reduction reaction (ORR), a hydrogen evolution reaction (HER), the removal of volatile organic compounds (VOCs), and CO preferential oxidation (PROX), etc. The plasma method allows thermodynamically and dynamically difficult reactions to proceed at low temperatures due to the activation of energetic electrons. Compared to conventional preparation methods, it has been proven to be a fast, facile, and environmentally friendly method for synthesizing highly efficient nanomaterials. The synthesized nanomaterials generally show enhanced metal–support interactions, small sizes of metal nanoparticles, specific metal structures, abundant oxygen vacancies, etc. Therefore, they exhibit high catalytic activity and stability in energy and catalytic applications. In spite of the growing interest in plasma for energy and catalytic nanomaterials, the synthesis mechanisms of nanomaterials using plasma still remain obscure due to the complicated physical and chemical reactions that occur during plasma preparation. A great deal of research is needed to better understand the controllable preparation mechanisms of the plasma method and to widen its application scope in synthesizing energy and catalytic nanomaterials.

Generally, solution plasma sputtering was used to prepared metal nanoparticles supported on carbon materials. Li et al. [1] used various organic quinolone (Q), aniline (A), and quinoline-aniline 1:1: mixed solution (QA) as nitrogen and carbon resources, synthesized different cobalt nanoparticles/nitrogen-doped carbon (Co/N-C) by solution plasma sputtering, and used as ORR electrocatalysts for zinc–air (Zn–Air) batteries. For ORR catalysts, N-doped species are crucial to electrocatalytic active sites. It is found that the as-obtained QA-Co/N-C sample with dominant quaternary-N and amino-N gave an onset potential of 0.87 V (vs. RHE) and a limit current density of 6.39 mA/cm^2^. When used in a primary aqueous Zn–Air battery, the QA-Co/N-C exhibited an open-current voltage of 1.43 V and the peak power density of 87 mW/cm^2^, which is comparable to those of the commercial 20 wt % Pt/C electrocatalyst. Moreover, QA-Co/N-C performed a stable galvanostatic discharge for 30,000 s at 20 mA/cm^2^ and showed a great potential to be a Pt-free ORR electrocatalyst.

With the help of gas plasma, active components and catalyst supports can be optimized for achieving great catalytic activity. Zhang et al. [2] employed gas plasma to activate Au/P25-As prepared by a modified impregnation method with different working gases (H_2_, Ar, O_2_, Air). The Au/P25-O_2_P catalyst activated by oxygen plasma showed excellent CO oxidation activity mainly due to the small size of gold nanoparticles and the high concentration of [O]_s_ species. The Au/P25-O_2_P exhibited a CO conversion of 100% at 40 °C, which is 30 °C lower than that of Au/P25-As catalyst. Liu et al. [3] etched nitrogen-doped carbon anchored by FeCo alloys (FeCo@NC) using dielectric barrier discharge (DBD) plasma in Ar atmosphere. Compared with FeCo@NC, the as-obtained DBD-FeCo@NC exposed more active sites, such as Fe/Co-N-C sites and enriched defect sites. The DBD−FeCo@NC performed an onset potential of 0.95 V as ORR electrocatalyst and an initial potential of 1.49 V as OER electrocatalyst, both of which are much better than those measured for FeCo@NC without Ar-plasma etching.

Gas plasma has also been proved to be a fast, facile, and green method for introducing groups to nanomaterials. Mi et al. [4] modified boron nitride nanosheets (BNNSs) by atmospheric pressure Ar+H_2_O low-temperature plasma initiated by bipolar nanosecond pulse DBD. The as-obtained plasma-modified BNNSs (P-BNNSs) contained nearly twice the content of surface hydroxyl than BNNSs. Moreover, the coating amount of silane coupling agent (SCA) on the surface of P-BNNSs increased by 45% more than the BNNSs, which enhanced the dehydration condensation reaction of P-BNNSs with SCA. Due to the P-BNNSs, the BN/epoxy resin (EP) insulating nanocomposites performed high thermal conductivity and high breakdown strength. Furthermore, some small molecule gases can be produced to exfoliate bulk particles and synthesize two-dimensional (2D) nanosheets via a plasma process. Zhang et al. [5] have successfully obtained 2D MoS_2_ nanosheets and 2D g-C_3_N_4_ nanosheets. Using H_2_/Ar plasma, the corresponding NH_3_ and H_2_S generated and expanded to the layers of bulk (NH_4_)_2_MoS_4_, while the bulk g-C_3_N_4_ was oxidized into corresponding CO_x_ and NO_x_ and generated 2D g-C_3_N_4_ nanosheets via air plasma. The prepared MoS_2_ and g-C_3_N_4_ nanosheets showed the thickness of 2–3 and 1.2 nm, respectively. They exhibited excellent photocatalytic activity due to the nanosheet structure, larger surface area, more flexible photophysical properties, and longer charge carrier average lifetime. It can be predicted that plasma as the environmentally benign approach provides a general platform for fabricating ultrathin nanosheet materials, which will greatly help the practical application and scientific research of 2D catalytic materials.

Unlike conventional preparation methods, which generally need excess toxic reducing chemical agents, plasma can be used to generate redox species with a green engineering. Fan et al. [6] prepared Ag nanoparticles supported on cotton fabric (Ag/Cotton) with high antibacterial activity against both the Gram-negative bacterium *E. coli* and the Gram-positive bacterium *B. subtilis* by a surface plasma at atmospheric pressure for the first time. Ag/Cotton exhibited remarkable unusual physical and chemical properties, and excellent antibacterial performance against a wide scope of pathogens. Xie et al. [7] synthesized aqueous gold nanoparticles (AuNPs) using a HAuCl_4_/sodium citrate solution via alternating the current plasma jet (A-Jet) and the pulse power driven plasma jet (P-Jet), respectively. Due to the high concentration of Cl^−^ and H_2_O_2_ in the A-Jet, the AuNP growth rate is more than 40 times faster than that in the P-Jet. Moreover, there is a broad size control range and a narrow AuNP size distribution in the A-Jet.

In DBD plasma, the electrode is crucial to the experimental apparatus as well as the packing materials, reactor, discharge power, etc. Li et al. [8] studied CO_2_ decomposition using DBD plasma with different metal foam electrodes, including Al foam, Fe foam, and Ti foam. For example, the Fe foam electrode exhibited more discharge area compared with the Fe rod electrode. The CO_2_ conversion using Fe foam electrode reached 44.84% (with a corresponding energy efficiency of 6.86%), which is much better than the 21.15% CO_2_ conversion reached using the Fe rod electrode (with a corresponding energy efficiency of 3.92%). Taheraslani et al. [9] investigated the deposits formed by CH_4_+Ar plasma processing in a packed bed reactor with packing materials including γ-alumina, Pd/γ-alumina, BaTiO_3_, silica-SBA-15, MgO/Al_2_O_3_, and α-alumina. Usually, the deposits mainly consist of carbon content (91 at. %) with the H/C molar radio around 1.7. Different from other packing materials, Pd/γ-alumina could restrain carbon-rich agglomerates due to the fast hydrogenation of deposit-precursors. Zhang et al. [10] measured vibrational energy distribution and electron energy distribution by high resolution temporal-spatial spectra emitted from the plasma, studied the characteristic evolution and discharge regimes transition of nanosecond pulsed DBD plasma, and distinguished the three main stages in the discharge, namely the streamer breakdown, the transition from streamer to diffuse regime, and the propagation of surface discharge on the plate electrode surface. It is important to develop plasma sources in material synthesis applications.

In summary, the papers published in this Special Issue include: plasma-assisted synthesized nanomaterials, a plasma-modified interface of nanomaterials, plasma-assisted catalysis, and the mechanism of plasma. Yu and co-workers [11,12] gave the brief overview of the advanced progress of plasma for energy and catalytic nanomaterials. The advanced nanomaterials with superior particle size and good dispersion could be synthesized by plasma-assisted preparation methods, including plasma enhanced atomic layer deposition technology, coaxial pulse arc plasma deposition, plasma sputtering, solution plasma sputtering, etc. Furthermore, gas plasma is employed to provide high energy state gas with free radicals, ions, and electrons, which could endow nanomaterials with surface active groups, heteroatom doping, surface etching, chemical oxidation/reduction, and high dispersed components. In addition, plasma could be directly used in catalytic reactions either with or without catalysts. Up until now, understanding the mechanism of plasma and precisely controlling the process of plasma has been a challenge.

## References

[1] Kim S., Park H., Li O.L. (2020). Cobalt Nanoparticles on Plasma-Controlled Nitrogen-Doped Carbon as High-Performance ORR Electrocatalyst for Primary Zn-Air Battery. Nanomaterials.

[2] Zhang J., Di L., Yu F., Duan D., Zhang X. (2018). Atmospheric-Pressure Cold Plasma Activating Au/P25 for CO Oxidation: Effect of Working Gas. Nanomaterials.

[3] Liu M., Yu F., Ma C., Xue X., Fu H., Yuan H., Yang S., Wang G., Guo X., Zhang L. (2019). Effective Oxygen Reduction Reaction Performance of FeCo Alloys In Situ Anchored on Nitrogen-Doped Carbon by the Microwave-Assistant Carbon Bath Method and Subsequent Plasma Etching. Nanomaterials.

[4] Mi Y., Gou J., Liu L., Ge X., Wan H., Liu Q. (2019). Enhanced Breakdown Strength and Thermal Conductivity of BN/EP Nanocomposites with Bipolar Nanosecond Pulse DBD Plasma Modified BNNSs. Nanomaterials.

[5] Zhang B., Wang Z., Peng X., Wang Z., Zhou L., Yin Q. (2019). A Novel Route to Manufacture 2D Layer MoS_2_ and g-C_3_N_4_ by Atmospheric Plasma with Enhanced Visible-Light-Driven Photocatalysis. Nanomaterials.

[6] Fan Z., Di L., Zhang X., Wang H. (2019). A Surface Dielectric Barrier Discharge Plasma for Preparing Cotton-Fabric-Supported Silver Nanoparticles. Nanomaterials.

[7] Xie P., Qi Y., Wang R., Wu J., Li X. (2019). Aqueous Gold Nanoparticles Generated by AC and Pulse-Power-Driven Plasma Jet. Nanomaterials.

[8] Li J., Zhai X., Ma C., Zhu S., Yu F., Dai B., Ge G., Yang D. (2019). DBD Plasma Combined with Different Foam Metal Electrodes for CO_2_ Decomposition: Experimental Results and DFT Validations. Nanomaterials.

[9] Taheraslani M., Gardeniers H. (2019). High-Resolution SEM and EDX Characterization of Deposits Formed by CH_4_+Ar DBD Plasma Processing in a Packed Bed Reactor. Nanomaterials.

[10] Zhang L., Yang D., Wang S., Jia Z., Yuan H., Zhao Z., Wang W. (2019). Discharge Regimes Transition and Characteristics Evolution of Nanosecond Pulsed Dielectric Barrier Discharge. Nanomaterials.

[11] Yu F., Liu M., Ma C., Di L., Dai B., Zhang L. (2019). A Review on the Promising Plasma-Assisted Preparation of Electrocatalysts. Nanomaterials.

[12] Li J., Ma C., Zhu S., Yu F., Dai B., Yang D. (2019). A Review of Recent Advances of Dielectric Barrier Discharge Plasma in Catalysis. Nanomaterials.

